# The Handwriting Is on the Wall

**Published:** 1906-07

**Authors:** 


### THE HANDWRITING IS ON THE WALL. IMPERIAL METHODS IN MEDICAL POLITICS.

It was Daniel who interpreted *mene, mene, tekel upharsin.* I paraphrase it: "Octopus, you have been weighed in the balance and found wanting" to boss things and run the "mersheen," and you are up against it.

By reference to another part of this issue it will be seen that a resolution introduced in the House of Delegates by H. O. Walker of Detroit, at the recent Boston meeting, asking for an investigation of the financial management of the Octopus was laid upon the table! Such is the autocratic power behind the House of Delegates. The House controls the Association; the Trustees the House, and the Secretary-Editor the Trustees. Witness a resolution adopted empowering the Trustees to change the place of meeting elected by the House of Delegates—if it didn't suit 'em. (Coe had a close call. They would gladly have cut out Portland had this come earlier.) Thus the power of nullifying the action of the House is put into the hands of the Trustees, which means the Secretary-Editor—The Czar! The fact that an investigation was shut off gives ground for suspicion that there is something—what is it?—that will not bear investigation. The 23,000 members and the 100,000 physicians not members have a right to know what is done with the $275,000 annual revenue and the $25,000 annual profit on the *Journal.* But they are denied this right, and are told to "shut up." That is about what McCurdy and McCall, and Perkins at first told the policy holders. They lost their heads. That is about what Louis XVI told the *rabble* when they asked for bread. That is about what Charles I. told Parliament. That is about what poor, weak little Nicholas has told the Douma. Charles lost his head, Louis lost his, and the Czar will lose his. The handwriting foretold the fall of Babylon. It foretells that of the Czar of all the medical profession.

In Texas we have a splendid organization with an enrolled membership close to 3000. Let it throw off the yoke, assert itself, and oil will be thrown on the troubled waters. Raise the Lone Star flag once more. Be we men and submit to be bossed and outraged? Already members are leaving the Octopus at the rate of one hundred a month. See Secretary's report elsewhere. "1208 were dropped during the year; 171 deaths, 619 resignations" (fifty a month every month) and the balance (presumably) for nonpayment of dues—same as resignations. True, there was a net gain of 4300. This was largely accessions from New York State, whose "new code" State Association has been whipped back or coaxed back. They bring in several thousand. This was a master stroke (?) of "the benevolent assimilation" policy, the homeopathic leveling process now going on,—"unifying the profession."

It will be remembered that in 1884 certain leading New York delegates favored consulting with homeopaths, and, in consequence, were refused admittance to the A. M. A. at the Milwaukee meeting, I think it was (in 1884). They split off and organized a separate State association, and were known as the "new code men." They were never afterwards represented in the A. M. A. They come back now and bring their tails behind them,—and all is lovely. And so goes on the leveling process, the loaf having been leavened with homeopathic leaven under the skillful manipulations of the redoubtable disciple of Hahneman, the Secretary-Editor of the Octopus.

But this big addition to the ranks was exceptional and can not occur again. In future the falling off will exceed the gains, and when the big Western State Medical Association now being organized by Jabez Jackson is inaugurated, the Western men will secede in a body. Then comes the seceding Southern States and the downfall of this modern Babel, where "medicine," homeopathy, faithhealing, osteopathy and voodoo are all represented and spoken.

